# An innovative small molecule for promoting neuroreparative strategies

**DOI:** 10.1039/c7ra11812k

**Published:** 2018-01-31

**Authors:** Nicola Antonio Colabufo, Marialessandra Contino, Mariangela Cantore, Francesco Berardi, Roberto Perrone, Annamaria Tonazzi, Lara Console, Maria Antonietta Panaro, Heli Savolainen, Gert Luurtsema

**Affiliations:** Dipartimento di Farmacia-Scienze del Farmaco, University of Bari “Aldo Moro” via Orabona 4 70125 Bari Italy nicolaantonio.colabufo@uniba.it; Biofordrug srl, Dipartimento di Farmacia-Scienze del Farmaco, University of Bari “Aldo Moro” via Orabona 4 70125 Bari Italy; CNR-IBIOM (Institute of Biomembrane, Bioenergetics and Molecular Biotechnologies) via Amendola 165/A 70126 Bari Italy; Department BEST (Biologia, Ecologia, Scienze della Terra), Unit of Biochemistry and Molecular Biotechnology, University of Calabria Arcavacata di Rende Italy; Dipartimento di Bioscienze, Biotecnologie e Biofarmaceutica, University of Bari “Aldo Moro” via Orabona 4 70125 Bari Italy; University of Groningen, University Medical Center Groningen, Department of Nuclear Medicine and Molecular Imaging Hanzeplein 1 9713 GZ Groningen Netherlands

## Abstract

In this study, a new regenerative strategy to treat several neurodegenerative diseases is suggested by the use of a multitarget approach induced by our small molecule, MC111. Considering the importance of P-gp and BCRP expression on stem cell differentiation and the involvement of TLR4 on neurodegeneration processes, we investigated the effect of MC111, belonging to our library of P-gp active compounds on: (i) TLR4 signaling; (ii) P-gp and BCRP activity and expression; (iii) neurite sprouting. The observed findings exerted by MC111, open a new scenario for a multitarget and regenerative approach in neurodegenerative diseases encouraging the *in vivo* evaluation of MC111 as new tool in neuroreparative medicine.

## Abstract 2

In this study, a new regenerative strategy to treat several neurodegenerative diseases is suggested by the use of a multitarget approach induced by our small molecule, MC111.

## Graphical abstract

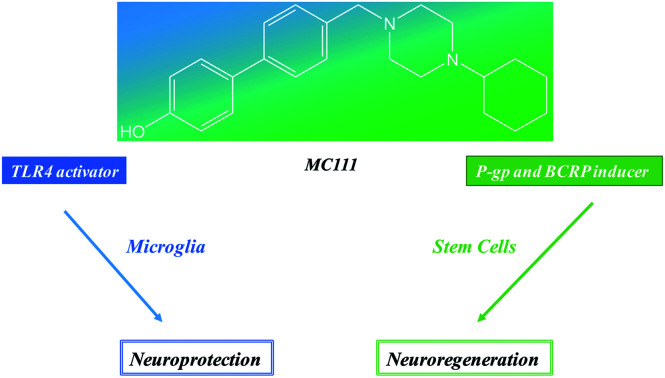

## Introduction

The challenge of this study is to suggest a new multitarget and regenerative approach disclosing new perspectives in neurodegenerative diseases. “Regenerative medicine” may provide an innovative treatment for many human disorders by the use of stem cells (SC). SC are a unique population of cells able to self-renew and differentiate into different cell types^1^ and they can be transplanted or manipulated *in vivo* to restore missing cells. Therefore, considering the capability of self-renewal and differentiation into a range of tissues of Neural Stem Cells (NSCs), SC-mediated therapy can be considered, at the present, the goal in cell replacement therapies in different pathological conditions such as in inflammation, neurodegeneration, immunomodulation and stimulation of endogenous recovery. Several reports demonstrated the expression of some ATP-binding cassette (ABC) transporters such as P-glycoprotein (P-gp, ABCB1) and Breast Cancer Resistance Protein (BCRP, ABCG2) in human neural stem/progenitor cells (hNSPCs).^2,3^ Moreover, the importance of ABC transporter expression and localization in NSCs on their differentiation and proliferation has been highlighted.^2^ Indeed, P-gp and BCRP expression represents a feature of proliferating stem cells and not of quiescent cells and their activities are involved in tissue regeneration mechanism, and in the protection from cell death under stress conditions.^2^

During the early developmental step, embryonic stem cells need a “protective barrier” due to the presence of ABC transporters pivotal in maintaining SC in an undifferentiated state; by contrast, their downregulation may induce an increased differentiation of NSCs.^2^

Indeed, Blood–Brain Barrier (BBB) endothelial cells produce soluble factors influencing the differentiation of NSPCs and in physiological conditions these factors, as substrates of ABC pumps, are effluxed by these transporters. When the expression of these proteins decreases, as in the early stage of Alzheimer's Disease (AD), these factors are not effluxed altering the microenvironment of stem cell niche and cellular homeostasis with functional alterations in neurogenesis.^1^ Therefore, since the expression of ABC transporters present in hNSPCs can be modulated, these proteins are the key for promoting hNSPCs self-renewal without differentiation. This link existing between ABC transporters present in NSCs can explain the potential of NSCs as therapeutic strategy in several brain disorders such as AD, Parkinson and Huntington's diseases through an endogenous activation. Moreover, an additional target identified in the early stage of AD is represented by Toll-Like Receptor 4 (TLR4);^4,5^ it is widely reported that microglia activation *via* TLR4 is closely associated to a number of neurodegenerative disorders including AD and ischemic stroke.^6^ In the senile plaques observed in the brain of AD patients, microglia is present in an activated state, and it is considered important in the pathogenesis of the disease. Microglia activation results in the overproduction of NO, oxygen free radicals, proteases, adhesion molecules and pro-inflammatory cytokines responsible for the degenerative process.^7^ AD patients and AD animal models display increased expression of CD14 (a co-receptor for TLR4), TLR4 and TLR2 ^8^ in response to the presence of Aβ and TLR4 mutation induces a decreased microglial activation, an increase in Aβ deposits and exacerbation of cognitive deficits.^4^ Therefore, as Aβ deposits occur, microglia is activated *via* TLR4 signaling to block Aβ deposition and to preserve cognitive functions from Aβ-mediated neurotoxicity.^9^ On the basis of these evidences, microglia activation *via* TLR4 in early stages of AD pathogenesis exerts a neuroprotective effect, and TLR4 pathways may be considered a potential therapeutic targets.

With all these aspects in mind, we hypothesized a multitargets drug, TLR4 inducer and P-gp/BCRP upregulating agent, simultaneously able to protect neurons by AD progression and in the meantime to activate the neuroreparative steps by maintaining stem cells in undifferentiated state. By a large screening of our P-gp/BCRP ligands library, we selected a small molecule named MC111.^10^MC111 profile has been deepened and the following activities have been studied: (i) the influence on TLR4 signaling; (ii) the activity towards P-gp and BCRP; (iii) the effect on neurites sprouting.

## Results

### MC111 influence on TLR4 signaling and on P-gp and BCRP expression

**Fig. 1:**
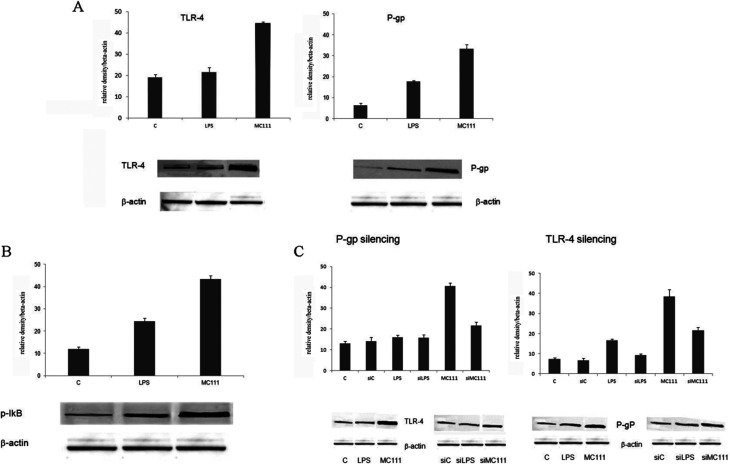
Histograms and densitometric WB analysis from SW480 cells. (A) TLR-4 and P-gp expression in SW480 cells ((C) unstimulated cells; (LPS) cells stimulated with 1 μg ml^−1^ LPS; (MC111) cells stimulated with 250 μM MC111. (B) Activation of NF-κB performed by analysis of active phosphorylated form (p-IκB). (C) Modulation of TLR-4 expression after P-gp silencing (left) and P-gp expression after TLR-4 silencing (right), respectively (siC: P-gp or TLR-4 silenced unstimulated cells; siLPS: P-gp or TLR-4 silenced cells stimulated with 1 μg ml^−1^ LPS; P-gp or TLR-4 silenced cells stimulated with 250 μM MC111).

MC111 was found able to increase TLR4 and P-gp expression (Fig. 1A) in SW480 human colon adenocarcinoma cells; TLR4 involved pathway has been deeply studied considering also the effect on other targets such as the nuclear factor NF-κB. TLR4 pathway involves the activation of NF-κB, as depicted in Fig. 1B; in unstimulated cells, NF-κB is sequestered in cytoplasm as inactive form by interacting with inhibitor of NF-κB (IκB) proteins. TLR4 stimulation by ligands leads to IκB phosphorylation with the release of NF-κB in the nucleus leading to the transcription of many pro-inflammatory genes encoding cytokines. In order to verify the existence of a link between ABCB1–TLR4 and MC111 activity, as depicted in Fig. 1C, P-gp (ABCB1) has been silenced and TLR4 expression detected in the presence of MC111; the ligand activity on P-gp expression has been evidenced after TLR4 silencing. The experiments demonstrated that MC111 activity on TLR4 and P-gp was reciprocally dependent from the expression of the two proteins.

Moreover, also MC111 activity profile towards BCRP has been investigated in MCF7 cells where BCRP, but not P-gp, is highly expressed.

### MC111 activity towards P-gp and BCRP

To assess how the expression and the activity of these proteins is influenced by MC111, the molecule has been probed in three cell lines (MCF7, hCMEC/D3 and Colo320 cells) in presence of radiolabeled P-gp and BCRP substrates, [^3^H]verapamil and [^11^C]MC150, respectively (unpublished results, formula depicted in supplementary materials). The substrates verapamil and MC150 are differently radiolabelled since verapamil is commercially available while MC150, belonging to our library, has been radiolabelled for *in vivo* PET analysis (studies in progress). The results demonstrated that MC111 increased the efflux of both substrates confirming that our ligand is an inducer of expression and also activity of the two pumps.

The immunostaining analysis on MCF7 cells demonstrated an increased BCRP expression already after 2 hours of MC111 exposition. In hCMEC/D3 cells, the same effect on BCRP expression was found after 72 h treatment with MC111.

The influence of MC111 on P-gp activity was assessed by subcellular fractionation experiments in Colo320 cells where the protein is highly present. In the presence of MC111, an increased [^3^H]verapamil efflux was observed. Moreover, also the efflux of a P-gp/BCRP radiolabeled substrate ([^11^C]-MC150) was increased by MC111 pre-treatment in Colo320 cells indicating induced BCRP activity.

### Neuroregeneration

**Fig. 2:**
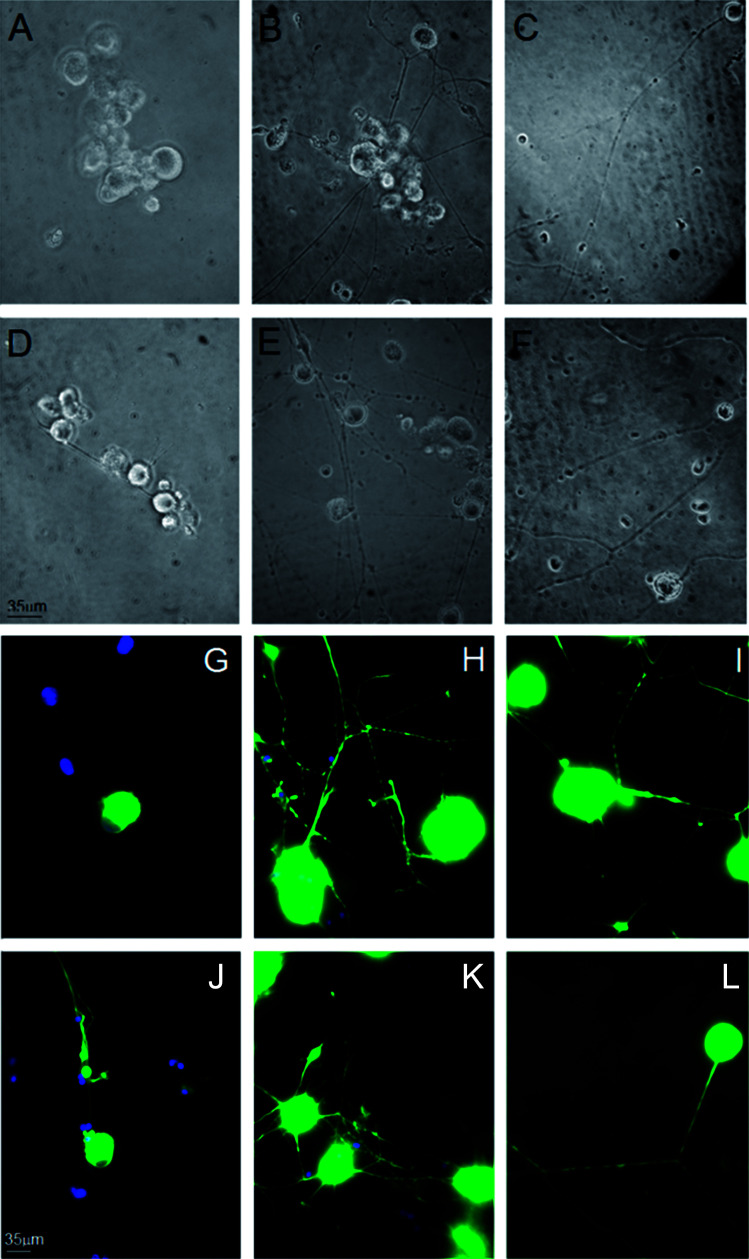
Representative images of Dorsal Root Ganglia (DRG) neurons after 72 h culture. (A–F) Phase contrast microscopy of DRG neurons cultured in the presence of BMS alone (A and D), BMS + 1 μg ml^−1^ NGF (B and E), BMS + 1 μM MC111 (C and F). (G–L) Immunocytochemical staining for βIII tubulin (FITC) of DRG neurons cultured in the presence of BMS alone (G and J), BMS + 1 μg ml^−1^ NGF (H and K), BMS + 1 μM MC111 (I and L).

We investigated the role of MC111 on dissociated primary sensory neurons with particular emphasis on the neurite growth changes. The effects of MC111 on Dorsal Root Ganglion (DRG) neurons in comparison to those cultured in a basic culture medium in the absence of NGF (negative control) or in the presence of NGF (positive control) was studied.^11^ DRG neurons culture was carried out following 3 different experimental conditions: Bottenstein and Sato's Medium (BSM) alone; BSM + 1 μg ml^−1^ NGF and BSM + 1 μM MC111. Neurite outgrowth from DRG living neurons after 72 h culture was observed under phase contrast microscope and images of the cells were digitized (Fig. 2A–F). From a qualitative analysis of the coverslips it appeared, that few cells cultured in BSM alone sprouted neurites (Fig. 2A and D) while the presence of either NGF (Fig. 2B and E) or MC111 (Fig. 2C and E) in the culture medium induced evident changes in neurite outgrowth of DRG neurons.

**Fig. 3:**
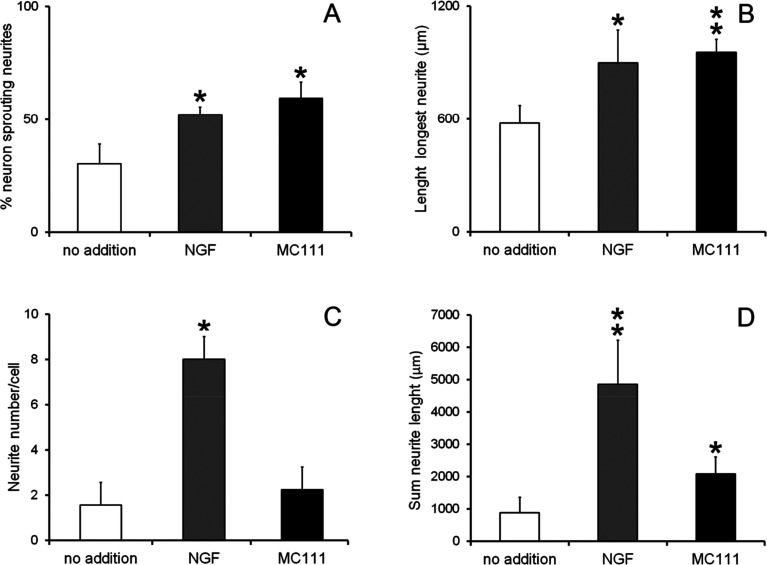
Quantification of DRG neurons neurite outgrowth after 72 h culture. BSM (white columns), BMS + 1 μg ml^−1^ NGF (grey columns), BMS + 1 μM MC111 (black columns). (A) Percentage of neuron sprouting neurites; (B) length of longest neurite; (C) neurite number per cell; (D) sum of neurite length. The values were the means ± SD from three experiments. *P < 0.05 **P < 0.01 *versus* control was estimated by Student's *t*-test.

Cell were then fixed and immunostained with βIII tubulin antibodies (Fig. 2G and J no addition; Fig. 2H and K NGF added; Fig. 2I and LMC111 added). Subsequent quantitative analysis of the DRG neurons was performed. Four parameters were measured: (1) % of cells containing neuritis respect to cells without neurites that resulted: 30% ± 8.7 (only BSM); 52% ± 3.5 (BSM + NGF); 59% ± 7.1 (BSM + MC111) (Fig. 3A). These results pointed out that both NGF and MC111 treatments induced a significant increase (*P* < 0.01) of neurons sprouting neurites in comparison to control. (2) Length of longest neurite: significant differences are evident between control neurons and neurons treated either with NGF or MC111; the length of the longest neurite was indeed similar between neurons treated with NGF or MC111 (Fig. 3B). (3) The number of neurites/cell: 1.6 ± 0.9 (only BSM); 8 ± 2.6 (BSM + NGF); 2.2 ± 0.8 (BSM + MC111). Only treatment with NGF was effective in inducing a significantly higher number of neurites respect to the control (Fig. 3C). (4) Total neurite length: 884 ± 467 μm (only BSM); 4848 ± 1363 μm (BSM + NGF); 2116 ± 494 μm (BSM + MC111). Both NGF and MC111 induced a significant increase in the sum of the total neurite length, that was indeed higher in the presence of NGF (Fig. 3D).

**Fig. 4:**
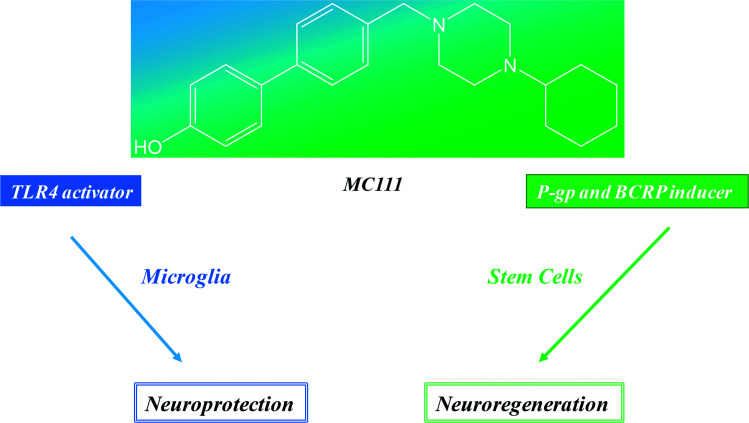
MC111 activities profile.

These results show that MC111 induces a relevant neurite sprouting with respect to the negative control but with different characteristics compared to NGF. Following treatment with NGF, a significant increase in total neurite number developed from each neuron was detected. The effect of MC111 was mainly represented by a moderate increase in neurite number and a great increase in the percentage of neuron spouting neurites with respect to the total neuron number, comparable with the increase obtained in the presence of NGF. The morphology of neurite sprouting from cells treated with MC111 is more similar to the physiological one, *i.e.* originating from the cellular body, that divides into two neurites (Fig. 2C, F, I and L). The observation that MC111 induces the growth of fewer but longer neurites in comparison to NGF, deserves particular attention in the perspective of peripheral nerve regeneration and repair (Fig. 4).

## Experimental

### Materials and method

#### MC111 influence on TLR4 signaling and on P-gp and BCRP expression

##### Cell cultures

To explore how MC111 influences TLR4 signaling we used SW480 cells since they express P-gp and can signal through TLR4.^12^ The human colon adenocarcinoma cell line SW480 (ICLC HTL99017-Interlab Cell Line Collection) was cultured on Leibovitz-15 medium supplemented with 10% fetal bovine serum, 100 U ml^−1^ penicillin, 100 μg ml^−1^ streptomycin and l-glutamine (2 mM; Life Technologies-Invitrogen).

Cultures were maintained at 37 °C in a humidified atmosphere containing 5% CO_2_ and expanded in tissue culture flasks (75 cm^2^; BD Biosciences), changing daily the medium.

The cells were seeded in six-well cell culture plates at 5 × 10^5^ cells per well and cultured to reach 80% confluency.

##### Cell treatments

Cell cultures were treated with lipopolysaccharide (LPS) from *Salmonella typhimurium* (Sigma-Aldrich) or with MC111 (concentration ranging from 500 nM to 250 μM). Preliminary experiments were performed in order to establish the optimal dose (1 μg ml^−1^) of LPS and MC111 (250 μM) and time of exposure to LPS (48 h). Untreated cells were used as control.

##### Treatment of cell cultures with small interfering RNA

Cell cultures (60–70% confluence) were submitted to P-gp or TLR-4 specific small interfering RNA (siRNA) using commercial kits (Santa Cruz Biotechnology) according to the manufacturer's protocol. Cells were incubated with 60–80 pmols of siRNA for 5–7 h at 37 °C, using a transfection reagent. Then, 1 ml of complete medium (2×) was added and cells incubated for 24 h. The day after, medium was replaced with fresh culture medium and cells were incubated for 48 h before experimental treatments. After this time, cells were submitted to LPS or MC111 treatment as indicated above.

##### Electrophoresis

After treatment as previously described, cells were washed twice in PBS, detached with ice-cold PBS, collected and centrifuged at 600*g* for 10 min. The supernatant was removed and the pellet was harvested and lysed by ice-cold lysis [1% (v/v) Triton X-100, 20 mM Tris–HCl, 137 mM NaCl, 10% (v/v) glycerol, 2 mM EDTA, 1 mM phenylmethylsulfonyl fluoride (PMSF), 20 μM leupeptin hemisulfate salt, 0.2 U ml^−1^ aprotinin (all from Sigma Aldrich)] for 30 min on an ice-bath. Lysate was centrifuged at 13 800*g* for 20 min at 4 °C. The protein concentration in the supernatant was spectrophotometrically determined by Bradford's protein assay and the lysate was subjected to SDS-PAGE (NuPage Electrophoresis System-Invitrogen). Protein samples were diluted 1 : 4 by NuPage LDS Sample Buffer 4× and 1 : 10 by NuPage Sample Reducing Agent (500 mM dithiothreitol (DTT) at 10× concentration) and underwent 10 min at 70 °C. Protein (30 μg lane-1) and prestained standards (Invitrogen) were loaded on 4–12% Novex Bis-Tris Midi gel 1.0 mm precast gels (Life Technologies-Invitrogen).

##### Western blotting

After electrophoresis, the resolved proteins were transferred from the gel to nitrocellulose membranes using iBlot Dry Blotting System A (Life-Technologies-Invitrogen). Then, membranes were blocked by PBS, (pH 7.2) containing 0.1% (v/v) Tween 20 and 5% (w/v) non-fat dried milk for 1 h and washed three times with 0.1% Tween 20-PBS (T-PBS). Specific proteins were detected using optimal concentration of primary antibodies, rabbit polyclonal anti human TLR-4 (1 : 200) or mouse monoclonal anti-human P-glycoprotein (MDR) (1 : 500). The binding of antibodies was detected with horseradish peroxidase (HRP)-conjugated goat anti-rabbit IgG followed by chemiluminescence detection (Pierce, Perbio, Rockford, USA). The β-actin protein level was used as a protein loading control in western blotting.

##### Densitometric analysis

The visualized bands obtained after immunoblotting experiments, were submitted to densitometric analysis using id image analysis software (Kodak Digital Science). β-actin was used for normalization of the immunoblotting products. Results were expressed as relative protein expression to β-actin.

##### Statistical analysis

Results were statistically examined by analysis of variance (one-way ANOVA) and a *p*-value < 0.05 was considered statistically significant.

#### MC111 activity on P-gp and BCRP

Commercially available chemicals were purchased from Sigma-Aldrich. [^3^H]-Verapamil (specific activity 80 Ci mmol^−1^, 1 mCi ml^−1^, solved in ethanol) was supplied by Biotrend. The primary anti-P glycoprotein antibody for mouse [JSB-1] and primary anti-BCRP/ABCG2 antibody for rat [BXP-53] were supplied by Abcam, UK or USA. The secondary antibody of P-gp anti-Mouse Alexa Fluor® 647 and secondary antibody of BCRP Anti-Rat Alexa Fluor® 488 were supplied by Abcam, UK or USA. Subcellular fraction buffer was prepared by the combination of the following reagents: 250 mM sucrose; 20 mM HEPES-hydroxyethyl piperazineethanesulfonic acid (pH 7.4) (Life Technologies); 10 mM KCl (Merck); 1.5 mM MgCl_2_ (Merck); 1 mM EDTA-ethylenediaminetetraacetic acid (Merck); 1 mM EGTA-ethylene glycol tetraacetic acid (Sigma-Aldrich); DTT (dithiothreitol) (Sigma-Aldrich); and protease inhibitor cocktail (Sigma-Aldrich).

Transwell™ 12-insert systems with tissue culture treated polycarbonate membrane, 8.0 μm pore size and 6.5 mm diameter inserts and Transwell™ 12-insert systems with tissue culture treated polyester membrane, 0.4 μm pore size and 12 mm diameter inserts were purchased from Corning Incorporated, USA. All reagents and chemicals used in this study are of analytical grade. Radioactivity measurements in cell studies were done using an automated gamma counter (Compugamma, LKB Wallac).

##### Cell lines

The colon cancer cell line of human origin, Colo320 and the human breast adenocarcinoma, MCF-7 (both kindly provided by the Medical Oncology Department of the UMCG, Netherlands) were cultured in Roswell Park Memorial Institute (RPMI) 1640 medium supplemented with fetal calf serum (10%) in a humidified incubator at 37 °C, 5% CO_2_ atmosphere. The cerebral microvascular endothelial cells of human origin, hCMEC/D3 (supplied by Institut Cochin, Paris, France), were cultured in RPMI 1640 supplemented with 10% fetal calf serum (FCS) Fetal Calf Serum (10%), penicillin (1%), hydrocortisone (1.4 μM), acid ascorbic (5 μg ml^−1^), HEPES (10 mM), bFGF (1 ng ml^−1^) in a humidified incubator at 37 °C, 5% CO_2_ atmosphere. The glioma cell line of rat origin C6 was cultured in Dulbecco's Modified Eagle Medium (DMEM) supplemented with 10% FCS in a humidified incubator at 37 °C, 5% CO_2_ atmosphere. Twice a week, the cells were harvested with trypsin and medium was changed.

##### Immunostaining of hCMEC/D3 and MCF7 cells

hCMEC/D3 or MCF7 cells were grown on gelatin-coated glass coverslips and exposed to MC111 for 24 and 72 h. After washing with cold PBS, cells were blocked with 1% PBS–BSA for 15 minutes; fixed with 4% paraformaldehyde (PFA) for 20 minutes; permeabilized with 0.2% Triton-X-100 for 10 minutes and blocked with 1% PBS–BSA for 15 minutes. The cells were incubated with 1% primary anti-P glycoprotein antibody or primary anti-BCRP/ABCG2 antibody in 1% PBS–BSA for 1 hour. The cells were then blocked in 15 minutes in 1% PBS–BSA and incubated in the dark with 0.02% secondary antibody for P-gp or BCRP for 30 minutes. Nuclear localization was also determined by staining with 1 μg ml^−1^ of DAPI. In between steps, the cells were washed 3 times with PBS. The expression of proteins was visualized by Leica DM6000B fluorescence microscope.

##### Subcellular fractions for detecting fraction-specific inducer uptake

Subcellular fractionation was performed at different concentration of inducer (5–300 μM) to establish the minimum concentration to reach the maximum uptake of [^3^H]-verapamil. Afterwards, this concentration was used to perform subcellular fractionation at different time points (20 min to 24 hours). The protocol for subcellular fractionation available on the website of Abcam®, https://www.abcam.com, was modified. At the time of use, 25 ml of stock subcellular fractionation buffer were taken and 25 μl of 1 mM dithiothreitol and 125 μl of protease inhibitor cocktail were added. The cells were treated with MC111 in a concentration range of 5–300 μM followed by addition of 0.2 μl [^3^H]verapamil (0.2 μCi). After 30 min incubation the medium was removed and the cells were harvested from the plate using trypsin and transferred into clean Eppendorf tubes containing RPMI 1640 medium for neutralizing the trypsin. To obtain the cell pellet, the cell suspension was centrifuged at 9000 rpm for 2 min. Supernatant was discarded and 500 μl of cold subcellular fractionation buffer was added to each cell pellet. Pellets were homogenized using 25 G needle and 1 ml syringe, distinct for each pellet, and kept on ice for 20 minutes. After homogenization, the suspension was centrifuged at 2500 rpm for 5 min. The supernatant was transferred into vials for liquid scintillation and counted on a γ-counter (cytoplasm fraction). 500 μl of the buffer was added to the cell pellet which was vortexed for 5 seconds, kept on ice for 10 minutes and centrifuged at 8000 rpm for 5 min. The supernatant was transferred into vials for liquid scintillation and was counted on a γ-counter (membrane fraction). The remaining pellets were also counted by liquid scintillation.

##### Drug transport assay using [^3^H]verapamil or [^11^C]MC150

Colo320 cells were seeded on microporous polycarbonate membrane filters (8.0 μm pore size, 6.5 mm diameter, Transwell™ 3422, Costar Corp., Cambridge, MD) at a density of 3 × 10^4^ cells per well. The cells were grown for 3 days. 1 h before the experiment, medium on both apical (100 μl) and basolateral side (600 μl) was replaced with RMPI complete medium. [^3^H]Verapamil 0.2 μl (0.2 μCi) or [^11^C]MC150 (15 μL–6.6 kBq μl^−1^) was added in the apical compartment following the addition of MC111 to yield a final concentration of 150 μM in the apical side. The cells were incubated at 37 °C at different times. After the incubation period, the medium from each side was removed and transferred to vials for liquid scintillation and samples were counted on a γ-counter. The ratio of apical to basolateral was calculated.
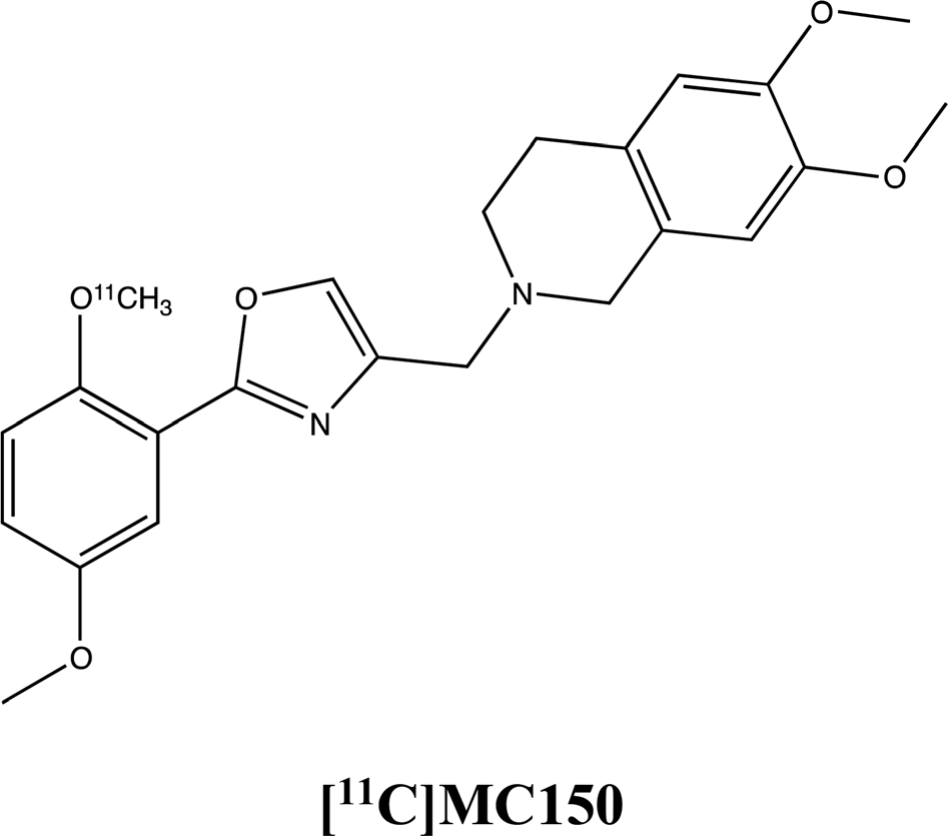


The chemistry and biology of this compound is already reported in our previous paper.^13^

#### Neuroregeneration

##### DRG neuron harvest

The animal experiments were performed according to the European Community guidelines for care and use of animals. In particular animals were housed in air-conditioned room with 12/12 h dark–light cycle, with food and water ad libitum, in the approved facility at the University of Bari. All animal procedures were performed in accordance with the Guidelines for Care and Use of Laboratory Animals of the “University of Bari (OPBA di Ateneo)” and the Italian Ministry of Health. Adult male Sprague-Dawley rat (age of 6–8 months) was anaesthetized by isoflurane before decapitation and the dorsal root ganglia (DRG) were gently pulled by its roots and harvested from all accessible levels using fine forceps. DRG were collected and placed on a Petri dish containing Ham's F12 medium at room temperature. The surrounding connective tissue and nerve roots of the DRG were removed under the microscope. Dissociated sensory neurons, from one rat for each assay, were prepared as previously reported.^14^ The DRG neurons were seeded on glass coverslips in 24 well plates pre-coated with 2 μg ml^−1^ laminin in modified Bottenstein and Sato's Medium (BSM; F12 medium containing 100 μM putrescine, 30 nM sodium selenite, 20 nM progesterone, 1 mg ml^−1^ BSA, 0.1 mg ml^−1^ transferrin, 0.01 mM cytosine arabinoside and 10 pM insulin). After 2 h BSM incubation (negative control), BSM supplemented with either NGF 1 μg ml^−1^ or MC111 1 μM was added to DRG culture which was incubated at 37 °C, 5% CO_2_ for 72 h.

##### Immunocytochemistry

Before immunostaining, living cells were examined and photographed under a Nikon DIAPHOT TS-100 microscope. Images were digitized with ×20 objective and a Nikon Digital Video Camera E995 (Coolpix 995). Subsequently the DRG neurons were fixed with 4% (w/v) PFA/PBS solution for 30 min at RT. After three PBS washes, the cells were permeabilized in 0.02% Triton X-100/PBS for 20 min. The cells were then incubated with 5% normal horse serum (NHS) (Vector Labs Inc.) for 1 h at RT to block non-specific binding sites. The blocking solution was replaced with PBS containing primary monoclonal mouse anti-βIII-tubulin antibody (Cell Signaling Technology) at 1 : 1000 dilution for 24 h at 4 °C. The cells were finally incubated with secondary FITC horse anti-mouse antibodies at 1 : 100 dilution (Vector Lab Ltd, UK) for 1 h at RT. The coverslips were mounted with Vectashield on glass microscope slides. Fluorescence was detected by an inverted Zeiss Axiovert 200 microscope equipped with epifluorescence. Cells were imaged with CoolSNAP HQ CCD camera (Roper Scientific, Trenton, NJ) using Metamorph software (Universal Imaging Corp., Downingtown, PA).

##### Neurite outgrowth analysis

Five independent culture experiments were carried out. Images of eight fields per well were taken. The number of cells sprouting neurites was determined by examining the field and counting cells with at least one neurite equivalent to the length of a cell body diameter. This number was expressed as a percentage of the total number of cells in the field. 8 bit images were loaded into ImageJ (version 1.47) and individual neurons, immunostained with anti-βIII-tubulin as described above, were manually traced with NeuronJ plugin (version version 1.4.2).^15,16^ The following parameters were analyzed: (1) % of sensory neurons spouting neuritis respect to all the sensory neurons present on the field; (2) length of longest neurite; (3) total number of processes grown in each field from all sensory neurons; (4) the total neurite length from all neurons in the field. Statistical analysis used to compare data from different groups was performed by Student's *t*-test. Values of *p* less than 0.05 or 0.01 were considered statistically significant.

## Conclusions

Neuroprotection and neuroreparative activities could be considered the two different faces of same medal where MC111 is the cohesive element. It represents a modern and smart drug able to hit different and synergistic targets of neurodegenerative pathways (TLR4, P-gp and BCRP induction) and exerts neuroregenerative effect although the molecular mechanism of the last should be investigated. Another important advantage of these results is that MC111 is the first small molecule able to give these combined effects and further its lead optimization is easy to carry out because of the simple structure of the ligand.

## Conflicts of interest

There are no conflicts to declare.

## Supplementary Material
